# Comparison of the efficacy of ROI-C cage with Zero-P device in anterior cervical discectomy and fusion of cervical degenerative disc disease: a two-year follow-up study

**DOI:** 10.3389/fsurg.2024.1392725

**Published:** 2024-06-03

**Authors:** Penghuan Wu, Sifan Yang, Yu Wang, Qiang Wu, Yingze Zhang

**Affiliations:** ^1^ The School of Medicine, Nankai University, Tianjin, China; ^2^ Department of Orthopaedics, Shao Guan First People's Hospital, Affiliated Southern Medical University, Guangdong, China; ^3^ Department of Orthopaedics, The Third Hospital of Hebei Medical University, Shijiazhuang, China; ^4^ Department of Medicine and Health, Chinese Academy of Engineering, Beijing, China; ^5^ NHC Key Laboratory of Intelligent Orthopeadic Equipment, The Third Hospital of Hebei Medical University, Shijiazhuang, China

**Keywords:** retrospective study, anterior cervical discectomy and fusion, cervical disc degenerative disease, ROI-C, Zero-P, 2-year follow-up

## Abstract

**Background:**

This study aimed to compare the clinical outcomes of Zero-P and ROI-C devices applied to anterior cervical discectomy and fusion (ACDF) surgery of cervical degenerative disc disease (CDDD).

**Methods:**

From January 2020 and December 2020, 56 patients with CDDD who underwent ACDF using Zero-P or ROI-C were included in this retrospective study. The outcomes included visual analogue scale (VAS) score, Japanese Orthopedic Association (JOA) score, neck disability index (NDI) score, Cobb angle, dysphagia, and bone fusion rate. Dysphagia was assessed using the Bazaz grading system. The comparison of outcomes between the two groups was based on the 2-year follow-up time point, which was defined as the last follow-up visit.

**Results:**

The Zero-P group included 16 males and 14 females, with a mean age of 56.2 (range, 35–65) years. The ROI-C group included 11 males and 15 females, with a mean age of 57.4 (range, 36–67) years. There was no significant difference in gender and mean age between the two groups. There were no significant differences in VAS score, JOA score, NDI score, Cobb angle, dysphagia, and bone fusion rate between two groups at the last follow up visit. In the Zero-P group, the duration of surgeries involving C3–4 or C6–7 segments was significantly longer than those including C4–5 or C5–6 segments (135.0 ± 19.0 vs. 105.6 ± 17.5 min, *P* < 0.05). In surgeries involving C3–4 or C6–7 segments, the operation time of ROI-C was significantly shorter than that of Zero-P (106.5 ± 19.5 vs.112.2 ± 20.5 min, *P* < 0.05). There were no significant differences in the dysphagia or cage subsidence rates between the Zero-P and ROI-C groups (*P* > 0.05). The Cobb angle in the last follow-up visit in the Zero-P group (24.4 ± 4.5°) was significantly higher than that in the ROI-C group (18.1 ± 2.3°) (*P* < 0.05).

**Conclusions:**

ACDF using ROI-C device showed an efficacy similar to the Zero-P device, as well as a shorter operation time for surgeries involving C3–4 or C6–7 segments. However, ROI-C could cause more loss of Cobb angle over time, which could lead to uncomfortable symptoms.

## Background

Cervical degenerative disc disease (CDDD) is a common spinal cord disorder affecting older people (1). Anterior cervical discectomy and fusion (ACDF) has been considered as the gold-standard treatment for symptomatic cervical spondylosis refractory to conservative management (2, 3). The plate-cage construct (PCC) applied to ACDF has become the standard method of anterior reconstruction to allow complete and immediate stability, thereby improving clinical outcomes (4). However, PCC is associated with complications, such as dysphasia, tracheoesophageal injury, and plate and screw loosening (5, 6). To minimize these complications, studies suggested some new anterior cervical interbody fusion and internal fixation systems, including the Zero-P and ROI-C devices (7, 8).

Both the Zero-P and ROI-C devices involve a cage, while their fixation styles differ. The former involves four screws, while the latter includes two clips, which can provide instant stability. Previous studies demonstrated that the Zero-P or ROI-C implant may achieve comparable outcomes with the PCC in terms of improving clinal outcomes and radiological parameters (9, 10). However, Zero-P or ROI-C implant have lower rates of surgical complications, such as dysphagia and cage subsidence (11, 12), because the two devices have a stable and compact “zero-profile” structure (1, 13).

Nevertheless, few studies have compared clinical outcomes and radiological parameters between Zero-P and ROI-C devices. Because of their similar structure, it is more necessary to compare their advantages and disadvantages for ACDF. The present study aimed to compare the two devices (Zero-P or ROI-C) for patients with CDDD undergoing ACDF in terms of better clinical outcomes and lower complication rates. The conclusion would provide valuable guidance for clinicians in selecting anterior cervical interbody fusion and internal fixation systems.

## Methods

### Study design and patients

This retrospective study included 56 patients with CDDD who underwent ACDF from January 2020 and December 2020. The inclusion criteria were as follows: (1) all patients had symptomatic CDDD and did not respond to at least 6 months of conservative treatment; (2) spinal cord or nerve root compression recently observed on magnetic resonance imaging (MRI); (3) consistency of clinical manifestations with radiological findings and physical examinations. The exclusion criteria were as follows: (1) treatment of ≥3 segments; (2) diagnosis of CDDD complicated with other spinal diseases (e.g., ossification of the posterior longitudinal ligament, hypertrophic ligamentum flavum, spinal tumors, cervical spinal trauma, spinal infections, severe osteoporosis, etc.); (3) a history of cervical spine injury or surgical intervention; (4) non-contiguous affected segments. Eligible patients were divided into two groups based on the type of implant used.

Patients were further subdivided according to the affected segments, which might influence the lenghth of operation time. Group A included C3–4 or C6–7 segments, and group B included C4–5 or C5–6 segments. The operation time was compared between groups A and B.

This study was approved by the Ethics Committee of the Third Hospital of Hebei Medical University (Shijiazhuang, China). The patients/participants provided their written informed consent to participate in this study. I confirm that all methods were performed in accordance with the relevant guidelines. All procedures were performed in accordance with the ethical standards laid down in the 1964 Declaration of Helsinki and its later amendments.

### Treatment process

**Figure 1:**
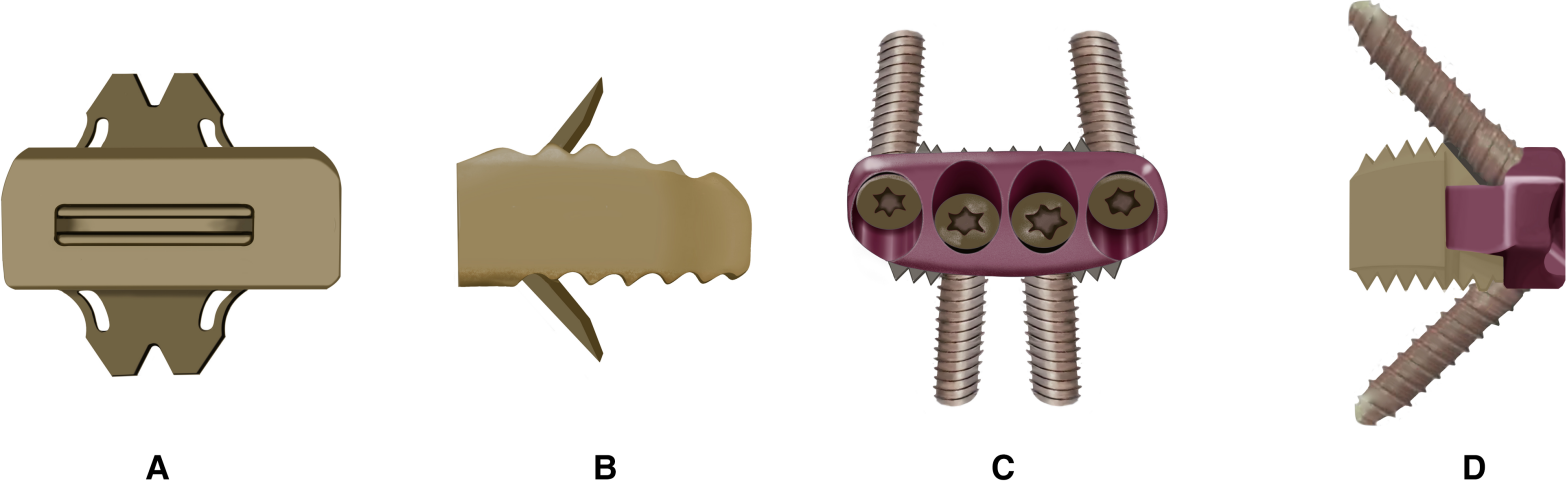
The schematic diagram of two devices. The ROI-C device: (**A**) anterior view; (**B**) lateral view; the Zero-P device: (**C**) anterior view; (**D**) lateral view.

According to medical records, all patients underwent surgery by the same senior surgeon. Preoperative x-ray (anterior-posterior, lateral, and flexion-extension), computed tomography (CT, sagittal reconstruction) and MRI were performed on each patient to confirm the affected segment(s). A Zero-P (DOUBLE MEDICAL, China) or ROI-C cage (LDR, French) (Figure 1) with an appropriate size (filled with allograft cancellous chips) was implanted into the segmental interbody region according to the trial spacers. Patients were asked to wear a neck brace for 4–6 weeks to avoid cervical flexion-extension.

### Data collection and definitions

All patients were followed up for at least 2 years. Clinical and radiological data were retrospectively collected preoperatively and at 24 months after surgery using medical records. The visual analogue scale (VAS) score was used to assess the level of neck and arm pain before surgery and at the last follow-up visit. The modified Japanese Orthopedic Association (JOA) scoring system was used to assess functional status before surgery and at the last follow-up visit. The recovery rate (%) at the last follow-up visit was calculated using the Hirabayashi's et al. method (14): (post-operative JOA score—pre-operative score)/(17—preoperative score) × 100%. The final recovery rate was classified as follows: ≤25%, poor; 25%–49%, fair; 50%–74%, good; and ≥75%, excellent. The neck disability index (NDI) was utilized to determine the degree to which neck pain was interfered with patients' ability to manage activities of daily living. Cervical lordosis (CL) was measured as the C2–C7 Cobb angle. The cervical range of motion (CROM) was defined as the sum of the C2–C7 Cobb angle on lateral x-ray images during flexion and extension. The disc height index (DHI) was the distance from the highest portion of the lower endplate of the cephalad vertebra to the closest portion of the upper endplate of the caudal vertebra. Subsidence was defined as the height loss >3 mm at any of the two measured disc heights (15). Patients were evaluated for dysphagia according to the subjective modified Bazaz grading system (16). Successful fusion was defined as <1-mm of interspinous motion on flexion-extension radiographs, with computed tomography follow-up if fusion status was indeterminate (17). All radiographs were read by two independent radiologists, and a third independent reading was conducted in case of disagreement.

### Statistical analysis

SPSS 25.0 software (IBM, Armonk, NY, USA) was used to perform statistical analysis. Categorical variables were expressed as number and percentile using frequency tables. Continuous variables were presented as mean ± standard deviation. For abnormally distributed data, logarithmic transformation was utilized to approximate the normal distribution. Paired *t-*test was utilized to compare significant differences between pre- and post-operative JOA score, VAS score, NDI score, CROM, C2–C7 Cobb angle, and DHI. Independent-samples *t*-test was used to compare intergroup differences in JOA score, VAS score, NDI score, CROM, C2–C7 Cobb angle, and DHI. For categorical variables, Pearson's *χ*^2^ test and Fisher's exact test were used. Differences were considered statistically significant at *P* < 0.05.

## Results

A total of 56 CDDD patients with a mean age of 56.6 (range, 34–65) years (27 men and 29 women) who underwent ACDF from January 2020 to December 2020 were retrospectively included. The number of cervical spondylotic radiculopathy was 42, cervical spondylotic myelopathy 10, and adjacent segment degeneration disease 4. The Zero-P group included 16 men and 14 women, with a mean age of 56.2 (range, 35–65) years. The ROI-C group involved 11 men and 15 women, with a mean age of 57.4 (range, 36–67) years. Demographic and baseline characteristics were comparable between the two groups (Table 1).

**Table 1:** Patient demographics.

Variable	Zero-P	ROI-C	Total	*P*
	(*n* = 30)	(*n* = 26)	(*n* = 56)	value
Age (years)	56.2 ± 5.4	57.4 ± 4.7	56.6 ± 5.1	0.281
Sex				
Male	16	11	27	0.801
Female	14	15	29	
Treated level				
C3–4	3	2		
C3–4 C4–5	2	3		
C4–5	5	3		
C4–5 C5–6	4	5		0.273
C5–6	8	7		
C5–6 C6–7	3	2		
C6–7	5	4		
Operative time (min)				
	119.3 ± 23.0	108.9 ± 19.7		0.077
Group A	135.0 ± 19.0	112.2 ± 20.5		0.010*
Group B	105.6 ± 17.5^#^	106.5 ± 19.5		0.892
Blood loss (ml)	56.5 ± 12.5	53.9 ± 15.7		0.326

* Significant difference between Zero-P and ROI-C using independent-samples *t*-test; *P* < 0.05.

^#^ Significant difference between group A and group B using independent-samples *t*-test; *P* < 0.05. Group A: including C3–4 or C6–7 level; Group B: neither including C3–4 or C6–7 level.

**Figure 2:**
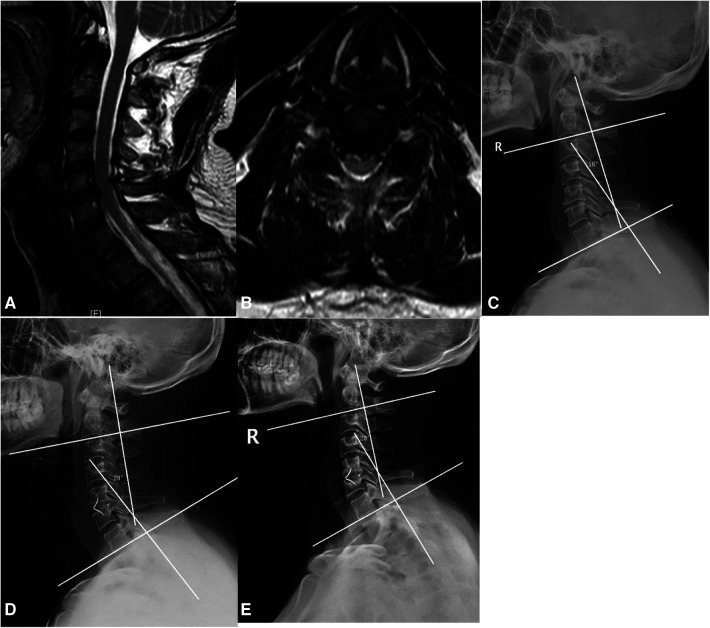
ACDF using ROI-C. (**A**) Sagittal MRI. (**B**) Axial MRI. (**C**) C2−7 cobb angle was 16°. (**D**) Immediately after surgery, C2−7 cobb angle was 29°. (**E**) C2−7 cobb angle decreased to 20° at the last follow-up visit.

**Figure 3:**
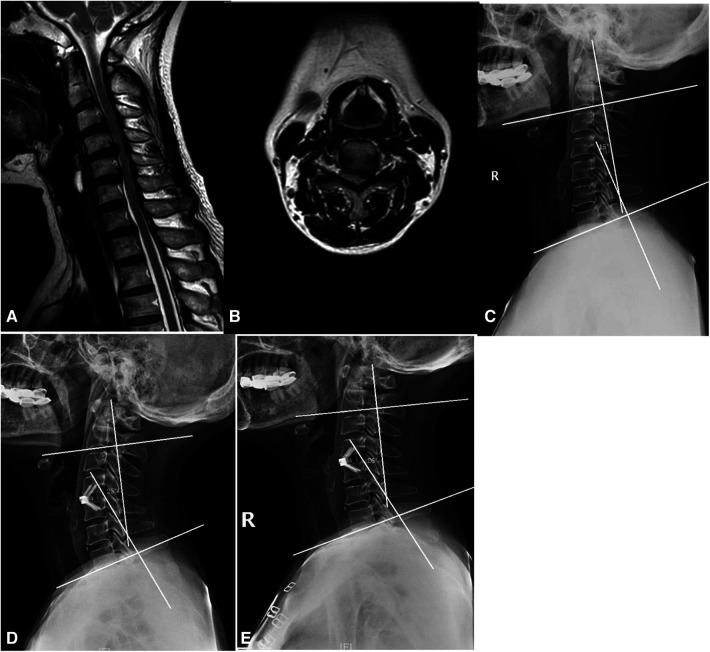
ACDF using Zero-P. (**A**) Sagittal MRI. (**B**) Axial MRI. (**C**) C2−7 cobb angle was 15°. (**D**) Immediately after surgery, C2−7 cobb angle was 26°. (**E**) C2−7 cobb angle was 25° at the last follow-up visit.

Patients were divided into the ROI-C (Figure 2) and Zero-P (Figure 3) groups. The two devices (Zero-P and ROI-C) achieved similar clinical and radiographical outcomes after ACDF at 2-year follow-up. There were no significant differences in age, gender, number of fused levels, operation time, or blood loss between the Zero-P and ROI-C groups (all *P* > 0.05, Table 1). However, the comparison of operation time between group A (C3–4 or C6–7 segments) and group B (C4–5 or C5–6 segments) revealed a significant difference (*P* < 0.05, Table 2). There were significant post-operative improvements for JOA score, VAS score, NDI score, DHI, and C2–7 Cobb angle between the two groups (*P* < 0.05, Table 2). However, the final follow-up Cobb angle in the Zero-P group (24.4 ± 4.5°) was significantly higher than that in the ROI-C group (18.1 ± 2.3°) (*P* < 0.05). In addition, 4 (4/56, 7.1%) patients with dysphagia and 1 (1/56, 1.7%) patient with cage subsidence were post-operatively identified. There was no significant difference between the Zero-P and ROI-C groups in the rates of dysphagia (*P* = 0.615) or cage subsidence (*P* = 0.464, Table 2). Immediately after surgery, 3 and 1 patients had mild and severe dysphagia, respectively. The above-mentioned 4 cases were improved within 3 months. At 2-year follow-up, radiographical examinations confirmed spinal fusion in all 56 patients, and the recovery rates in the Zero-P and ROI-C groups were 76.6% and 76.9%, respectively (Table 3). Not neurological or vascular complications or wound infection were perioperatively detected.

**Table 2:** Comparison of surgical outcomes between Zero-P and ROI-C.

Outcome	Zero-P		ROI-C	
	Preop	Last follow-up	Preop	Last follow-up
VAS score	7.3 ± 2.2	1.9 ± 1.6*	6.9 ± 2.6	2.1 ± 1.8*
JOA score	7.8 ± 2.4	15.2 ± 2.8*	15.2 ± 2.8*	15.8 ± 3.1*
NDI score	52.5 ± 7.6	12.6 ± 1.9*	54.8 ± 9.7	12.8 ± 2.3*
CROM (°)	38.4 ± 9.3	27.4 ± 10.6*	39.1 ± 9.6	28.1 ± 8.5*
C2–C7				
Cobb angle (°)	11.6 ± 7.4	24.4 ± 4.5*	11.4 ± 7.2	15.1 ± 2.3*†
DHI (mm)	5.4 ± 1.2	6.8 ± 1.1	5.6 ± 0.8	6.5 ± 1.3
Dysphagia		3/30		1/26
Cage				
subsidence		0/30		1/26
Bone fusion		30/30		26∕26

CROM, cervical range of motion; DHI, disc height index; JOA, Japanese orthopaedic association; NDI, neck disability index; VAS, visual analogue scale.

* Significant difference (*P* < 0.05) between preop and last follow-up with independent-samples *t*-test.

† Significant difference (*P* < 0.05) between Zero-P and ROI-C with independent-samples *t-*test or Pearson's *χ*^2^ test.

**Table 3:** Odom's criteria for postoperative outcomes.

Efficiency	Zero-P	ROI-C	*χ* ^2^	*P*-value
Excellent	8	7		
Good	15	13		
Fair	5	5		
Poor	2	1		
Success rate	23/30 (76.6%)	20/26 (76.9%)	0.001	0.982

No Significant difference in successful treatment rate between the two groups using Pearson's *χ*^2^ test; *P* > 0.05. Last follow-up CT value of Zero-P and Roi-C.

## Discussion

This is a study that compared the ROI-C cage and Zero-P device in ACDF. The present study showed that the two devices possess the similar efficacy, however the ROI-C device may have the lower incidence rates of hoariness and dysphagia at the cervical segment near the submandibular or sternal region, the ROI-C device was also easy to operate at these segments.

Multiple studies suggested that ROI-C and Zero-P devices have a high fusion rate (13, 18), which is consistent with the results of the present study. The teeth of ROI-C and screws of Zero-P are fitted into the vertebral body, making the cage more stable (11). Using a ROI-C device, fused vertebrae was achieved at mean time of 4.5–6.9 months (11). However, the 2-year bone fusion rate of Zero-P device was 93.9%. In the present study, the two groups achieved the bone fusion rate of 100% at the 2-year follow-up. Due to retrospective design of the study, the fusion rate was not recorded accurately before 2-year. The locking system could ensure excellent primary stability of implant and promote fusion (11).

The present study revealed that the dysphagia rate in the Zero-P group was higher than that in the ROI-C group, while no significant difference was found. Operation time in the Zero-P group was longer than that in the ROI-C group. Patients who undergo longer time ACDF may be at a greater risk of post-operative dysphagia (19). Longer time surgeries require a longer duration of pulling the esophagus in the supine position.

Importantly, it was noted that the operation time in the group A (C3–4 or C6–7 segments) was 20 min shorter for ROI-C than Zero-P, and the operation time for Zero-P in group A was longer than that in group B (C4–5 or C5–6 segments). There were no significant differences between the two devices when the surgery did not involve C3–4 or C6–7 segments. These results are in accordance with previously reported findings (20). As the ROI-C device only needs to be vertically hammered into the cervical vertebra through a cage without obstruction by jaw or sternum, it can therefore save time if surgery includes C3–4 or C6–7 segments (8). Accordingly, it might be a better choice to use the ROI-C device if surgery includes C3–4 or C6–7 segments. This surgical challenge may also be solved using a universal screwdriver (8). During ACDF surgery of Cervical Adjacent Segment Disease, the screws of Zero-P device might be blocked by the screws of plate-cage construct at the same vertebrate, while ROI-C might avoid this unexpected result for the shorter clips.

The results of the present study showed that the Cobb angle significantly improved for both devices, with no significant difference between the two groups. The post-operative improvement of Cobb angle could be related to the size of cage, which was implanted by a surgeon adopting the same surgical standard. However, the present study revealed that the Cobb angle in the ROI-C group was smaller than that in the Zero-P group. Cho et al. compared the trend in changes of Cobb angle at 2 years after implantation of Stand-Alone cage and Zero-P device and concluded that maintenance of normal cervical curvature was inferior with Zero-P device (21). The decreased anterior Cobb angle in the ROI-C group could be related to the fact that cross-sections of the two fixed clips were small and the shear force was larger. Although the clips did not easily regress, they may become deeper (21). Conversely, the Zero-P device was tightly screwed to the fusion cage. As they were firmly screwed into the vertebra, the screws were unlikely to loosen (22). In addition, 5 patients' post-operative Cobb angles were smaller than their preoperative Cobb angles. CL loss or kyphosis development could lead to cervical degeneration and cause pain, dysfunction, and other uncomfortable symptoms (23). Thus, other complications, such as CL in the long run, should be considered when cage devices are applied to ACDF.

Overall, the Zero-P and ROI-C devices showed a similar efficacy for ACDF. Both devices restored the normal physical lordosis of cervical vertebral and foraminal height and could be used in surgical discectomy. However, surgeons should take operative segments and personal proficiency into account during selection of cage devices.

The present study had several limitations. Firstly, because it was a single-center study, its sample size was limited, we would conduct a multi-center study in the future. Secondly, the follow-up time was relatively short. Thirdly, due to poor compliance of patients, we can not confirm specific time point of fusion before two years. Finally, the fusion rates were mainly obtained using radiographs, and fusion rates might be therefore overestimated.

## Conclusions

In summary, there was no significant difference in clinical outcomes for ACDF between Zero-P and ROI-C devices. ROI-C is an potential alternative device for ACDF surgeries involving C3–4 or C6–7 segments. However, ROI-C may cause more Cobb angle loss over time, which may cause uncomfortable symptoms. Additional large-scale biomechanical studies on cage stability with a longer follow-up time should be conducted in the future.

## Data Availability

The raw data supporting the conclusions of this article will be made available by the authors, without undue reservation.
